# Epidemiology and ‘big data’

**DOI:** 10.1007/s10654-017-0294-3

**Published:** 2017-08-17

**Authors:** Bruno H. Stricker

**Affiliations:** Department of Epidemiology, Erasmus Medical Center, PO Box 2040, 3000 CA Rotterdam, The Netherlands

In the past 70 years, epidemiology has gone through a substantial development of both methodology and more sophisticated analyses with determinant- and disease-specific elements. Despite the discussion earlier in this journal as to whether the duality of epidemiology’s embrace of both research methods and content-based topics is a positive development [1, 2], it shows at least that epidemiology as a discipline is able to re-invent itself and to adapt to the rapid scientific evolution which currently takes place. Whereas the methodological ‘toolkit’ keeps its solid basis in cohort- and case-control designs, determinant-oriented genetic epidemiology and pharmacoepidemiology, and disease-oriented cardiovascular and infectious diseases epidemiology are examples of branching within one discipline. While the start of epidemiology had its main focus on hypothesis-testing concerning the association between determinants and disease as well as risk estimation, the importance of hypothesis-generating studies is increasing. A nice example is the technique of genome-wide association studies which led to an explosion of discoveries in genetic epidemiology, also by a new model of collaborative science [3]. However, genes remain while drugs come and disappear again from human society. This makes pharmacoepidemiology an important hypothesis-generating and -testing vacuum cleaner of the pharmaceutical market. Elsewhere in this journal, Chia-Cheng Lai et al. [4] reviewed ‘sequence symmetry analysis’ as a technique for detecting adverse drug events by utilizing computerized claims data. Data mining techniques such as this one are considered of increasing importance. There are two reasons for that development. First, the enormous expansion of information technology in the past 30 years facilitated the easy access to huge amounts of healthcare information. In the stone age of pharmacovigilance and pharmacoepidemiology, a new adverse reaction not discovered in clinical trials was usually reported in the first years of marketing by intelligent medical observers with an open eye for new and unexpected events in their patients [5]. The more relevant of these reports were actively published in medical journals as ‘short reports’ or ‘letters to the editor’. The most serious ones were occasionally investigated in relatively small hospital-based cohort studies or population-based case-control studies with the aim to confirm signals from adverse event reports and calculate the magnitude of a risk increase. Such studies were expensive and time-consuming because data had to be collected and collated *de novo*. Nowadays, expensive multi-center studies on adverse reactions to drugs such as the International Agranulocytosis and Aplastic Anemia Study [6] and the International Primary Pulmonary Hypertension Study Group [7] would probably no longer be performed in that way. The more and more easy access to healthcare information in databases in most developed countries in the world is too efficient to omit. Second, there is the growing risk aversion in Western countries and fear for litigation. Consequently, increasingly strict legislation for obtaining a marketing authorization by regulatory authorities such as the FDA and the European Medicines Agency EMA have forced the pharmaceutical industry to demonstrate their active surveillance of literature, and signal generating activities. How far these requirements go, can be read in EMA guidelines for signal management [8]. Also, jurisdiction regarding adverse reactions in the past, especially in the U.S.A., demonstrates the important consequences of inadequate adverse reaction management [9].

Is this all good news? After all, everybody will recognize the importance of early detection of adverse reactions and use of healthcare information which is already available. However, we should not lose sight of the other side of the coin. Datamining of healthcare data generates many false-positive signals which have to be checked because the strict legislation and jurisdiction on adverse events, negligence and medical errors [10], in combination with risk aversion might force society to investigate every signal. Unfortunately, the efficiency of datamining is probably much lower than reporting of adverse events by intelligent observers [11] and will drain resources from important hypothesis-testing research with higher a priori chances of confirmation. As for hypothesis-testing, one might question whether the abundant availability of automated healthcare information will lead to a decline of the number of *de novo* epidemiological field studies in pharmacoepidemiology. This would be a loss because healthcare information may be very prone to selection and information bias while every studied intervention will suffer from confounding by indication. Information in such databases is healthcare-driven and determinants are not gathered for everyone and in the same way for all members of a population. For instance, potential confounders such as smoking or BMI are often not registered. Nevertheless, there is a strong tendency to use such information by health care insurance companies and other health care bodies for guidelines and pharmaco-economic modeling. Increasing employment of healthcare databases for post-authorization studies is too efficient to discourage but the potential misclassification of disease and co-factors should not be taken too lightly and lead to a card house of facts mixed with fictions. Big data are as attractive as nuclear energy to some of us. Keeping validity on board seems to me as being a greater challenge than dualities within our discipline.
